# Supplementary material for: Comparative Analysis of Actaea Chloroplast Genomes and Molecular Marker Development for the Identification of Authentic Cimicifugae Rhizoma
Source: Plants (Basel). 2020 Jan 27;9(2):157. doi: 10.3390/plants9020157 (PMC7076663; doi:10.3390/plants9020157)
Supplement: Supplementary file 1 [file plants-09-00157-s001.pdf]

SUPPLEMENTARY MATERIALS

Comparative Analysis of *Actaea* Chloroplast Genomes and Molecular Marker Development for the Identification of Authentic Cimicifugae Rhizoma

Inkyu Park, Jun-Ho Song, Sungyu Yang and Byeong Cheol Moon*

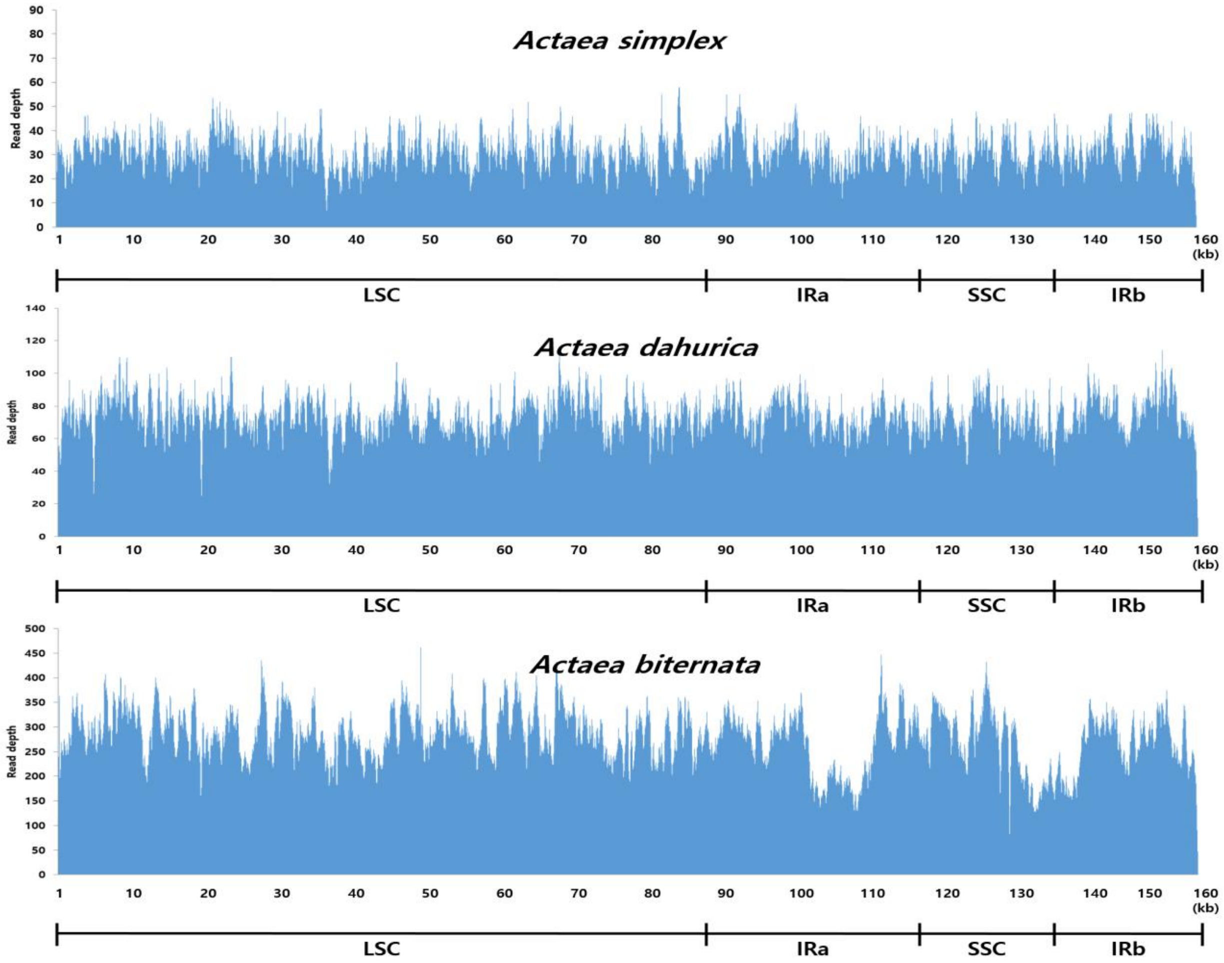

Figure S1: Validation of the complete chloroplast (cp) genomes of three *Actaea* species, *A. simplex*, *A. dahurica*, and *A. biternata* using raw read mapping. LSC, large single copy region; SSC, small single copy region; IRa, inverted repeat a; IRb, inverted repeat b.

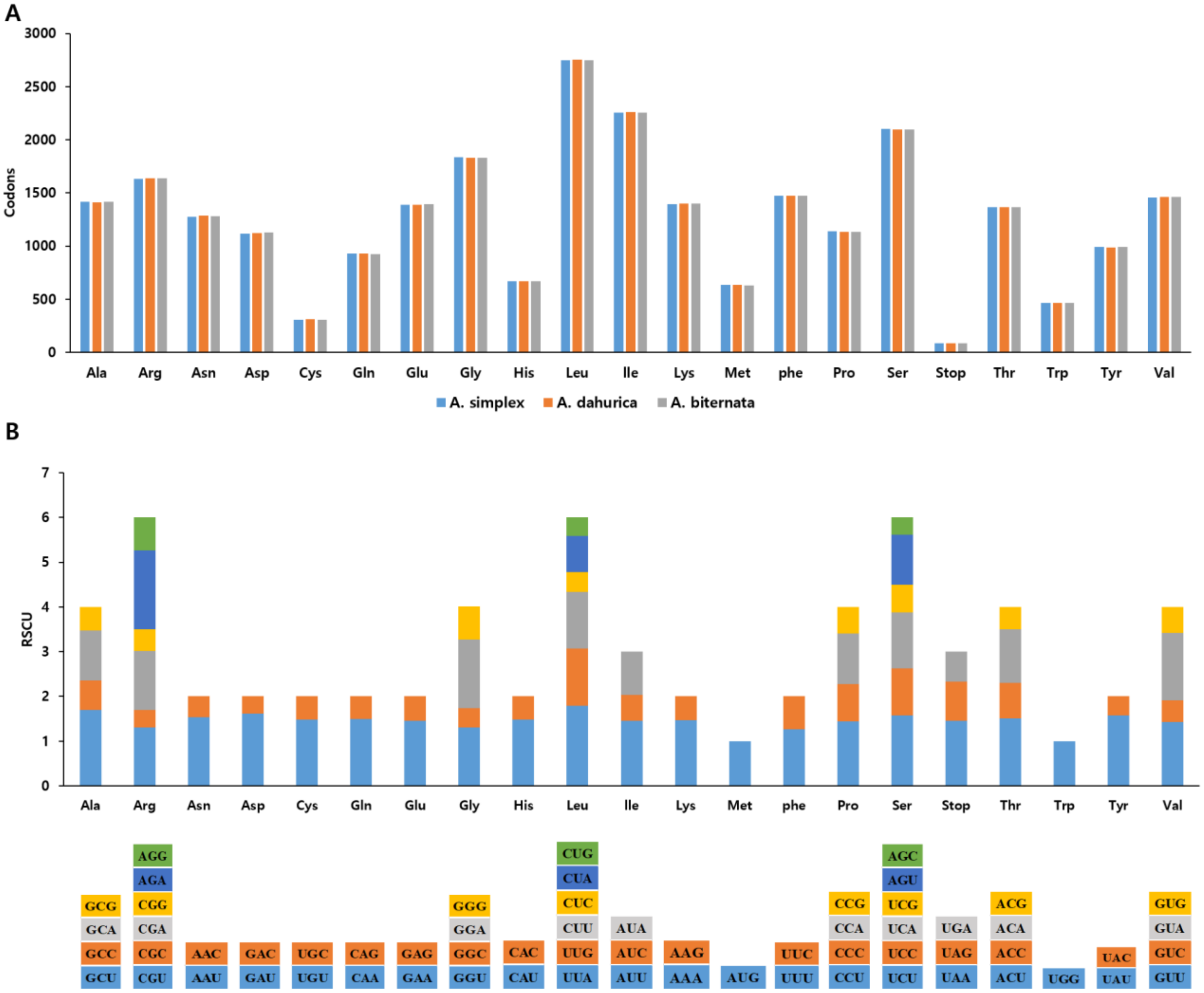

Figure S2: Codon frequencies and RSCU (relative synonymous codon usage) values of three *Actaea* species, *A. simplex*, *A. dahurica*, and *A. biternata*. (A) Amino acid frequencies in protein-coding genes. (B) RSCU values of 20 amino acids and stop codons in 78 protein-coding genes.

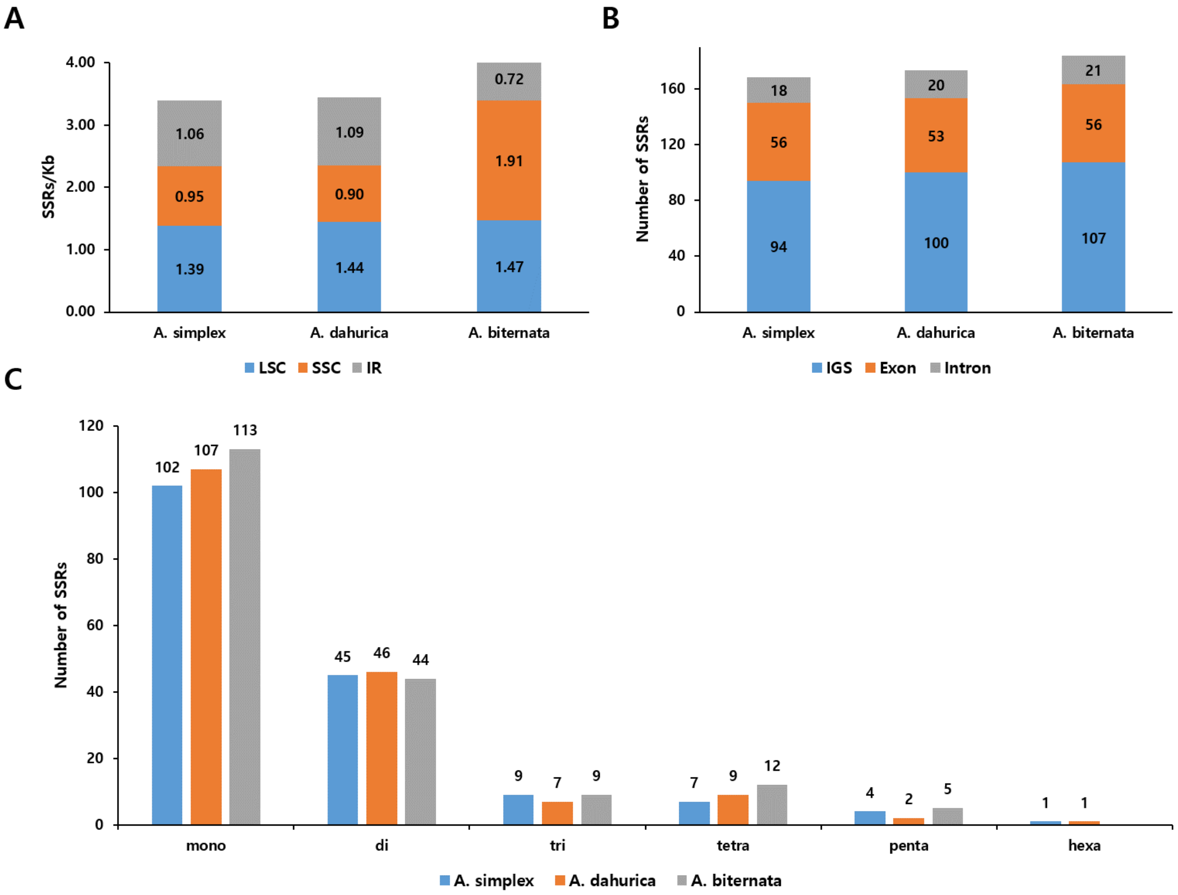

Figure S3: Distribution of simple sequence repeats (SSRs) in cp genomes of three *Actaea* species. (a) Number of SSRs per unit length. (b) Distribution of SSRs in exons, introns and intergenic spacer (IGS) regions. (c) Distribution of different types of SSRs.

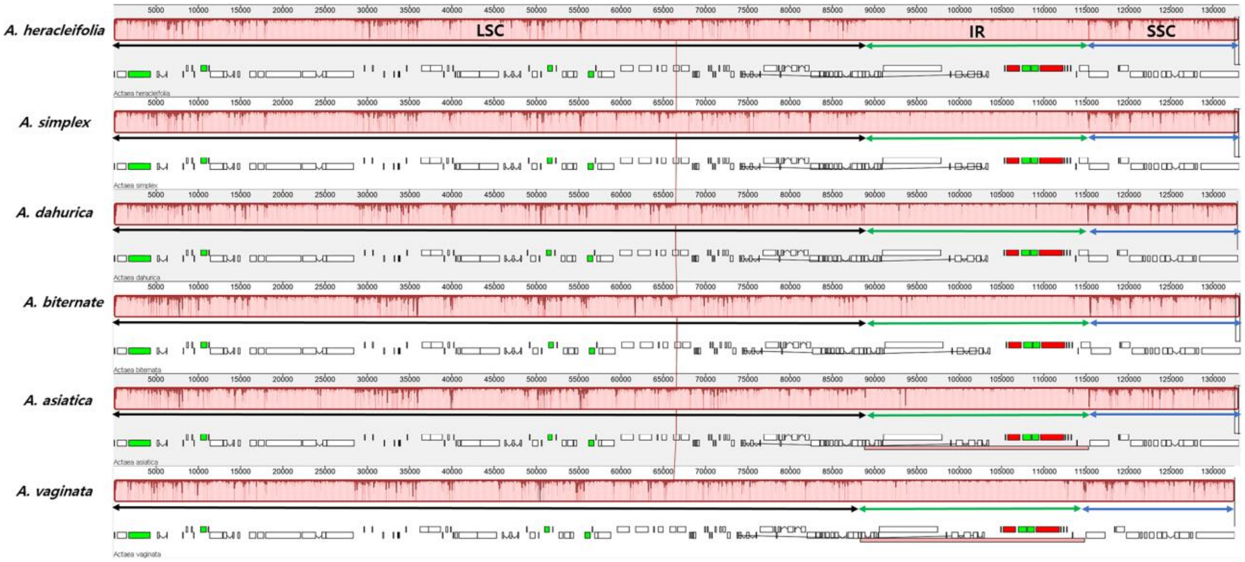

Figure S4. Comparison of complete cp genomes of six *Actaea* species using the MAUVE algorithm. Local collinear blocks colored in red indicate syntenic regions, while histograms within each block represent the degree of sequence similarity. Arrows indicate the large single copy (LSC), inverted repeat (IR), and small single copy (SSC) regions. Green color blocks means tRNAs with intron. And red color block indicates rRNAs.

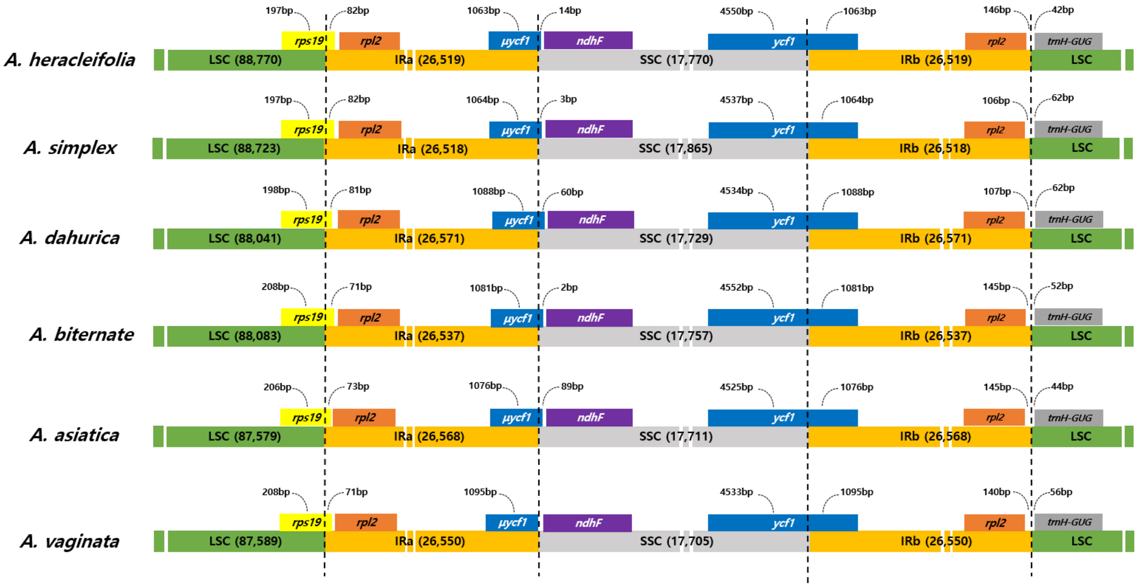

Figure S5: Comparison of the position of junctions between the large single copy (LSC) region, inverted repeat (IR) regions (IRa and IRb), and small single copy (SSC) region in the cp genomes of six *Actaea* species.

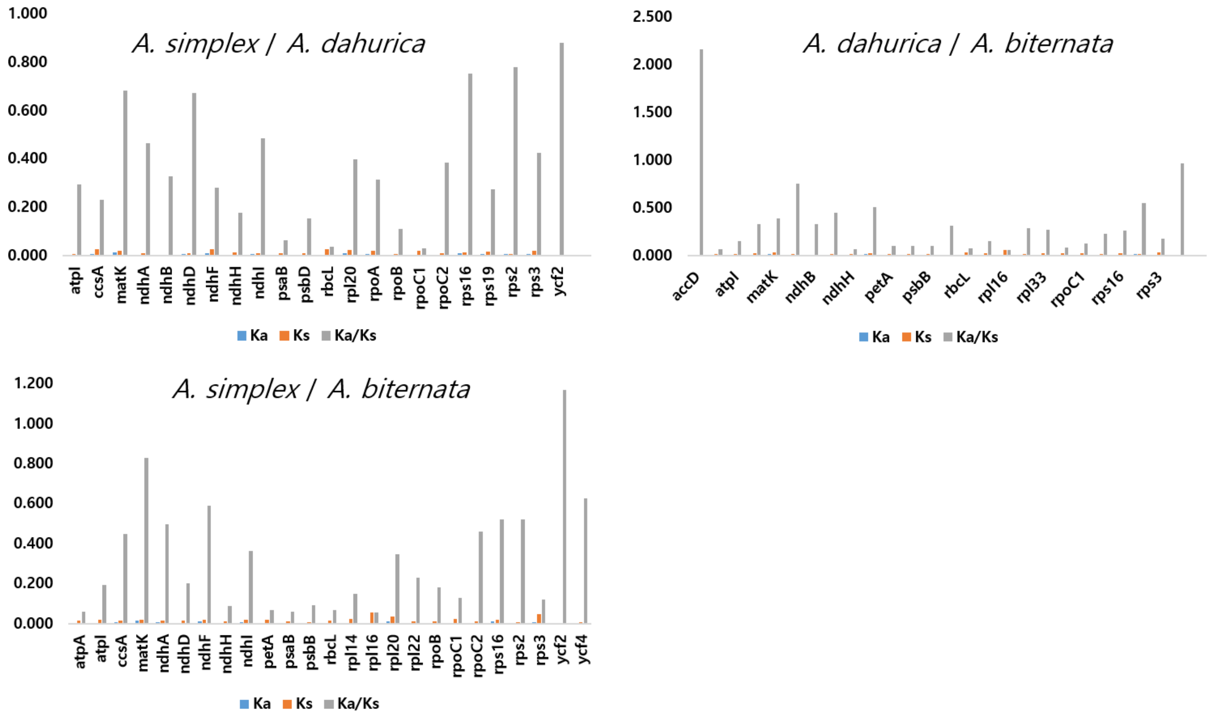

Figure S6: Comparison of non-synonymous substitution rate (Ka), synonymous substitution rate (Ks) and Ka/Ks ratio of 67 conserved protein-coding genes among the cp genomes of *A. simplex*, *A. dahurica*, and *A. biternata*. Blue and orange bars indicate Ka and Ks values, respectively, while gray bars indicate Ka/Ks ratios. Genes with Ka or Ks values of 0 are not shown.

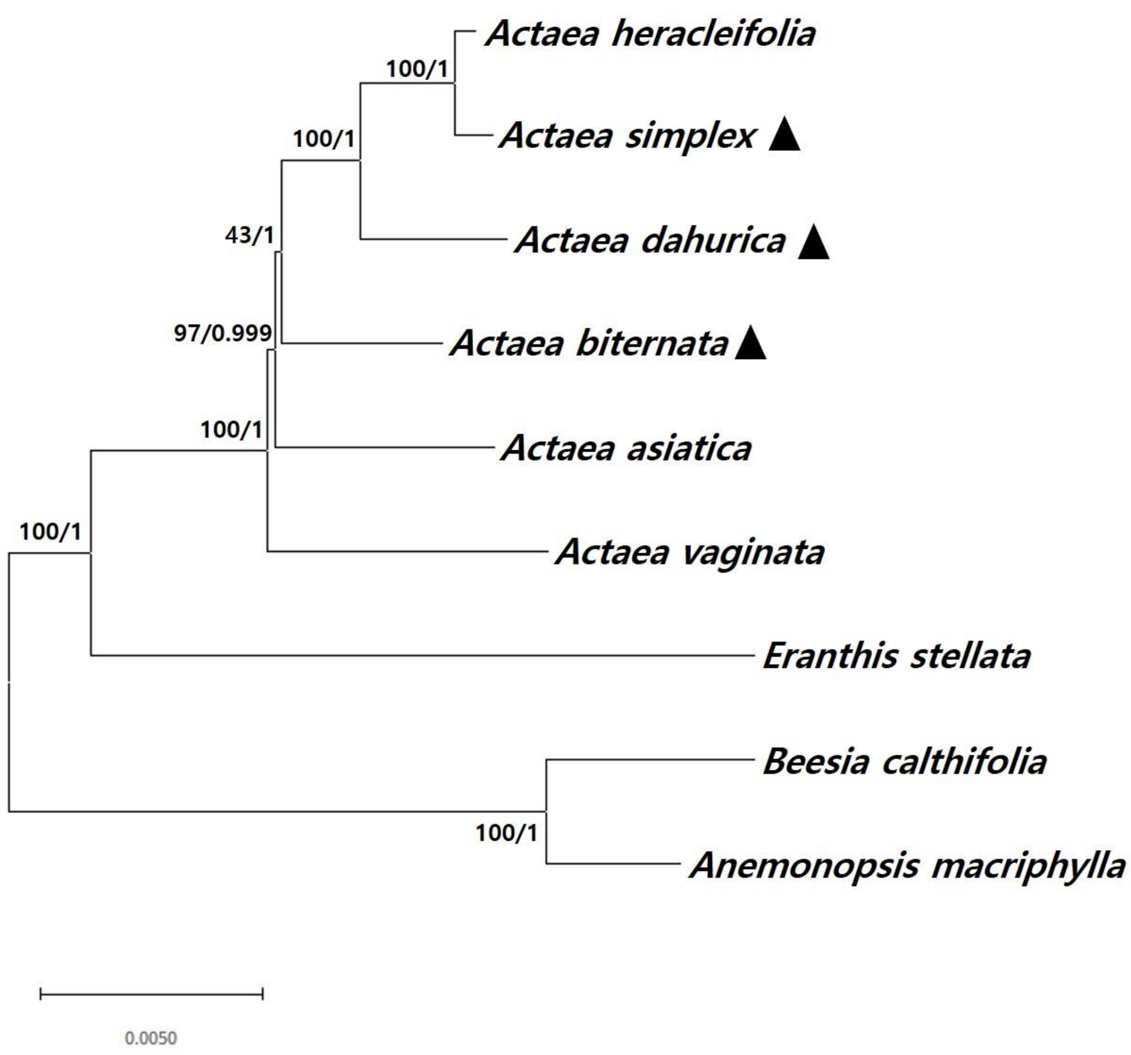

Figure S7: Phylogenetic analysis of nine species within the tribe Cimicifugeae. The phylogenetic tree was constructed with the maximum likelihood (ML) method and Bayesian Inference (BI) using whole cp genome sequences of six *Actaea* species and one *Eranthis* species. *Beesia calthifolia* and *Anemonopsis macrophylla* were used as outgroups. ML topology is shown with bootstrap (BS) values (%) and BI posterior probability (PP) values at each node. Black triangles indicate the cp genomes of three *Actaea* species investigated in this study.

Table S1: Raw read and assembly information of the chloroplast (cp) genomes of three *Actaea* species.

	A. simplex	***A. dahurica***	***A. biternata***
Number of input reads	7,455,916	7,348,266	8,077,362
Number of trimmed reads	6,415,201	6,240,189	7,211,294
Total raw bases (bp)	2,196,684,451	2,184,464,508	2,347,534,148
Trimmed bases (bp)	1,500,015,928	1,462,941,174	1,706,161,420
Number of aligned reads	22,304	52,247	194,903
Coverage (×)	31.51	74.67	280.52
cp genome length (bp)	159,624	159,523	159,789

Table S2: Primers used for PCR-based sequence validation of cp junctions in three *Actaea* species.

Primer name	Primer sequence (5′→3′)	Junction†
LI_F	CCCGGGACCAAGTTACGATT	LSC/IRa
LI_R	AGTTTGATTCTTCGTCGCCGT	
IS_F	ACTTCCGAAACGAAGGGGAC	IRa/SSC
IS_R	AAATATGTAGGCGGAGGGCG	
SI_F	CGTTGCTCAGGTTGTTTCGT	SSC/IRb
SI_R	GGATCCACCGAAGAAGACCC	
IL_F	AACCCTGTAGACCATCCCCA	IRb/LSC
IL_R	GTTCAATTCCCGTCGTTCGC	

†LSC, large single copy region; SSC, small single copy region; IRa, inverted repeat a; IRb, inverted repeat b.

Table S3: Information of PCR-based sequencing.

Species	Junction†	PCR product size (bp)	Start position (bp)	End position (bp)	Identity (%)
A. simplex	LSC/IRa	202	88,697	88,898	100
	IRa/SSC	240	114,964	115,203	100
	SSC/IRb	558	132,791	133,348	100
	IRb/LSC	364	159,324	63	100
A. dahurica	LSC/IRa	207	88,625	88,831	100
	IRa/SSC	467	114,922	115,388	100
	SSC/IRb	556	132,663	133,218	100
	IRb/LSC	379	159,219	74	100
A. biternata	LSC/IRa	213	88,921	89,133	100
	IRa/SSC	517	115,201	115,717	100
	SSC/IRb	562	132,950	133,511	100
	IRb/LSC	374	159,489	73	100

†LSC, large single copy region; SSC, small single copy region; IRa, inverted repeat a; IRb, inverted repeat b.

Table S4: Genes present in the cp genomes of three *Actaea* species.

**Gene group**	**Gene name**
Photosystem I	*psaA, B, C, I, J, ycf3*†, *ycf4*
Photosystem II	*psbA, B, C, D, E, F, H, I, J, K, L, M, N, T, Z*
Cytochrome b6/f	*petA, B*§, *D*§, *G, L, N*
ATP synthase	*atpA, B, E, F*§, *H, I*
Rubisco	*rbcL*
NADH oxidoreductase	*ndhA*§, *B*§,¶, *C, D, E, F, G, H*¶, *I, J, K*
Large ribosomal subunit proteins	*rpl2, 14, 16*§, *20, 22, 33, 36*
Small ribosomal subunit proteins	*rps2, 3, 4, 7*¶, *8, 11, 12*†, ¶, ¥, *14, 15, 18, 19*
RNA polymerase	*rpoA, B, C1*§, *C2*
Protein-coding genes with unknown function	*ycf1, ycf2*
Other genes	*accD, ccsA, cemA, clpP*†, *matK*
Ribosomal RNA (rRNA) genes	*rrn16*¶, *23*¶, *4.5*¶, *5*¶
Transfer RNA (tRNA) genes	*trnA-UGC*§, ¶, *trnC-GCA, trnD-GUC, trnE-UUC, trnF-GAA, trnfM-CAU, trnG-GCC,trnG-UCC, trnH-GUG, trnI-CAU, trnI-GAU*§, ¶,*trnK-UUU, trnL-CAA, trnL-UAA, trnL-UAG, trnL-CAA*¶, *trnM-CAU, trnN-GUU*¶, *trnP-UGG, trnQ-UUG, trnR-ACG*¶, *trnR-UCU, trnS-GCU, trnS-GGA, trnS-UGA, trnT-GGU, trnT-UGU, trnV-GAC*¶, *trnW-CCA, trnY-GUA*

§Genes containing a single intron.
†Genes containing two introns.
¶Genes with two copies in inverted repeats (IRs).
¥Trans-splicing gene.

Table S5: Gene intron in cp genomes of three *Actaea* speceis.

	Gene name	Location†	Exon I (bp)§	Intron I (bp)§	Exon II (bp)§	Intron II (bp)	Exon III (bp)
1	*trnk-UUU*	LSC	37	2544 (2551, 2543)	35		
2	*rps16*	LSC	40	892 (892, 907)	221		
3	*trnG-UCC*	LSC	23	694 (688, 688)	48		
4	*atpF*	LSC	144	728 (723, 727)	411		
5	*rpoC1*	LSC	435	754 (759, 754)	1620		
6	*ycf3*	LSC	126	713 (715, 715)	226	757 (744, 745)	155
7	*trnL-UAA*	LSC	35	508 (505, 502)	50		
8	*trnV-UAC*	LSC	38	581 (582, 581)	37		
9	*rps12*	LSC	232		26		114
10	*clpP*	LSC	69	812 (813, 807)	291	646 (636, 633)	246
11	*petB*	LSC	6	803 (800, 799)	642		
12	*petD*	LSC	8	723	496		
13	*rpl16*	LSC	9	999 (998, 1004)	402		
14	*ndhB*	IR	777	700	756		
15	*trnI-GAU*	IR	42	941	35		
16	*trnA-UGC*	IR	38	803 (803, 800)	35		
17	*ndhA*	IR	551	905 (920, 917)	541		

†LSC, large single copy region; SSC, small single copy region; IR, inverted repeat.

§Numbers in parentheses represent the length of corresponding sequences in *A. dahurica* and *A. biternata*.

Table S6: Tandem repeats detected in the cp genome of *A. simplex*.

A. simplex	Position†	Repeat unit length (bp)	Repeat unit sequence	Repeat number	Location§
1	IGS (*trnK-UUU*, *rps16*)	23	TTTTTTCGTTATTTATTTTA TAT	2	LSC
2	IGS (*rps16*, *trnQ-UUG*)	12	ATATATATTATA	2	LSC
3	IGS (*rps16*, *trnQ-UUG*)	16	AGATTGTTCAAGACAA	2	LSC
4	IGS (*rps16*, *trnQ-UUG*)	8	GTAATAAA	3	LSC
5	IGS (*trnS-GCU*, *trnG-UCC*)	13	AATTGAATAAAAA	2	LSC
6	IGS (*atpH*, *atpI*)	19	TTCATTAGTCTGAATCCTT	2	LSC
7	IGS (*petN*, *psbM*)	13	TACGAGAATCCAG	2	LSC
8	IGS (*petN*, *psbM*)	15	TTTTACAAATATGAA	2	LSC
9	IGS (*trnS-GGA*, *rps4*)	17	ATACTGCAATAATATGA	2	LSC
10	IGS (*trnL-UAA*, *trnF-GAA*)	25	TCTTTCACAAAAGGATCG TGGGAGA	2	LSC
11	Exon (*accD*)	15	AATGACTATTCATCT	2	LSC
12	IGS (*psaI*, *ycf4*)	17	TATGTAGATATCCATAG	2	LSC
13	Intron (*rpl16*)	15	TTAATGCTTTATTAC	2	LSC
14	Exon (*ycf2*)	21	TCTTTTTGTCCAAGTCGCT TC	3	IR
15	Exon (*ycf2*)	18	TATTGATGATAGTGACGA	3	IR
16	IGS (*rps12*, *trnV-GAC*)	16	ATTTTATTGATTTATT	2	IR
17	IGS (*rrn5*, *trnR-ACG*)	15	AATATGAAAAAAAAA	2	IR
18	IGS (*ndhF*, *trnL-UAG*)	21	ACAAATAGAACAAGTAA AAAA	2	SSC
19	IGS (*ndhF*, *trnL-UAG*)	20	TTTATTCGTATTAATATTA A	2	SSC
20	IGS (*ndhE*, *ndhG*)	22	AATTAACTAAAATATAAT ATAA	2	SSC
21	Exon (*ndhG*)	12	GTATTTAGTATT	2	SSC

†IGS, intergenic spacer region.

§LSC, large single copy region; SSC, small single copy region; IR, inverted repeat.

Table S7: Tandem repeats detected in the cp genome of *A. dahurica*.

A. dahurica	Position†	Repeat unit length (bp)	Repeat unit sequence	Repeat number	Location§
1	IGS (*trnH-GUG*, *psbA*)	12	AATATATACAAA	2	LSC
2	IGS (*atpH*, *atpI*)	21	TAATACAAGCATGAATAC AAA	2	LSC
3	IGS (*atpH*, *atpI*)	19	TTCATTAGTCTGAATCCTT	2	LSC
4	IGS (*petN*, *psbM*)	13	TACGAGAATCCAG	2	LSC
5	IGS (*petN*, *psbM*)	15	TTTTACAAATATGAA	2	LSC
6	IGS (*psbM*, *trnD-GUC*)	8	AATCATAT	3	LSC
7	IGS (*trnE-UUC*, *trnT-GGU*)	8	ATTTCTAT	3	LSC
8	IGS (*trnE-UUC*, *trnT-GGU*)	15	TTCTTCAATTCAACT	2	LSC
9	IGS (*psbZ*, *trnG-GCC*)	18	ATATATT	2	LSC
10	IGS (*ycf3*, *trnS-GGA*)	17	ATACTGCAATAATATGA	2	LSC
11	IGS (*trnL-UAA*, *trnL-UAA*)	25	TCTTTCACAAAAGGATCG TGGGAGA	2	LSC
12	IGS (*ndhC*, *trnV-UAC*)	13	TATATTTTATTTGA	2	LSC
13	IGS (*ndhC*, *trnV-UAC*)	10	TATTATTATC	2	LSC
14	IGS (*ndhC*, *trnV-UAC*)	19	GTACAGTACAAATATAGT A	2	LSC
15	IGS (*atpB*, *rbcL*)	16	TTATTATTCATTATTA	2	LSC
16	IGS (*rbcL*, *accD*)	15	AATGACTATTCATCT	2	LSC
17	IGS (*rbcL*, *accD*)	17	TTTAGTTTATAGTAATC	2	LSC
18	IGS (*psaI*, *ycf4*)	17	TATGTAGATATCCATAG	2	LSC
19	Intron (*rpl16*)	15	TTAATGCTTTATTAC	2	LSC
20	Exon (*ycf2*)	21	TCTTTTTGTCCAAGTCGCT TC	3	IR
21	Exon (*ycf2*)	18	TATTGATGATAGTGACGA	4	IR
22	IGS (*rps12*, *trnV-GAC*)	16	ATTTTATTGATTTATT	2	IR
23	IGS (*rrn5*, *trnR-ACG*)	13	AATATGAAAAAAA	2	IR

†IGS, intergenic spacer region.

§LSC, large single copy region; SSC, small single copy region; IR, inverted repeat.

Table S8: Tandem repeats detected in the cp genome of *A. biternata*.

A. biternata	**Position†**	**Repeat unit length (bp)**	**Repeat unit sequence**	**Repeat number**	**Location§**
1	Intron (*rps16*)	9	TATAGTATA	2	LSC
2	IGS (*rps16, trnQ-UUG*)	21	ATAATAATACATATTTAT	2	LSC
3	IGS (*rps16, trnQ-UUG*)	28	TTTAGAGATAAGGAATAT ATAATATAAA	2	LSC
4	IGS (*rps16, trnQ-UUG*)	17	AATAGAAAGAAAACAAA	2	LSC
5	IGS (*atpH, atpI*)	19	TTCATTAGTCTGAATCCTT	2	LSC
6	IGS (*petN, psbM*)	15	TTTTACAAATATGAA	2	LSC
7	IGS (*trnT-GGU, psbD*)	25	TATTATCTATATAACGATA TGGATA	2	LSC
8	IGS (*psbZ, trnG-GCC*)	24	TATAAATATATAATGTTAT AGTTA	2	LSC
9	IGS (*ycf3, trnS-GGA*)	17	ATACTGCAATAATATGA	2	LSC
10	IGS (*ndhC,trnV-UAC*)	13	ATTTTTTTATTAT	2	LSC
11	IGS (*rbcL, accD*)	19	AGTTCCTATATTTATTTTG	2	LSC
12	IGS (*rbcL, accD*)	46	TATAATATATATAGACTTG ATCTAGATATAAAAAA GATCTTATCTT	2	LSC
13	IGS (*rbcL, accD*)	15	AATGACTATTCATCT	2	LSC
14	IGS (*psaI, ycf4*)	17	TATGTAGATATCCATAG	2	LSC
15	Exon (*ycf2*)	18	TATTGATGATAGTGACGA	4	IR
16	IGS (*rps12, trnV-GAC*)	16	ATTTTATTGATTTATT	2	IR
17	IGS (*rrn5, trnR-ACG*)	12	AATATGAAAAAA	2	IR
18	IGS (*ccsA, ndhD*)	15	TAGGAATAGCATATA	2	SSC
19	IGS (*ndhE, ndhG*)	22	AATTAACTAAAATATAAT CTAA	2	SSC
20	Exon (*ycf1*)	15	TTTTTATTTTCACTA	2	SSC

†IGS, intergenic spacer region.

§LSC, large single copy region; SSC, small single copy region; IR, inverted repeat.

Table S9: List of *Actaea* accession numbers used in this study.

A. heracleifolia	Collection site	Voucher number
1	Mungoksodo-dong, Taebaek-si, Gangwon-do	KIOM201501013910
2	Daeseong, Hwagae, Hadong, Gyeongsangnam-do,	KIOM201401009497
3	Ungyo, Bangnim, Pyeongchang, Gangwon-do	KIOM201401010685
4	Singi-ri, Jinbu, Pyeongchang, Gangwon-do	KIOM201401011037
A. simplex		
5	Gohan, Gohan, Jeongseon, Gangwon-do	KIOM201401010623
6	Ungyo, Bangnim, Pyeongchang, Gangwon-do	KIOM201401010683
7	Gohan, Gohan, Jeongseon, Gangwon-do	KIOM201401010894
8	Taegi, Dunnae, Hoengseong, Gangwon-do	KIOM201401011006
A. dahurica		
9	Mungoksodo, Taebaek, Gangwon-do	KIOM201501013910
10	Jeoksang, Muju, Jeollabuk-do	KIOM200601000057
11	Gohan, Gohan, Jeongseon, Gangwon-do	KIOM201401010625
12	Mungoksodo, Taebaek, Gangwon-do	KIOM201501013910
A. biternata		
13	Aewol, Jeju, Jeju-do	KIOM201001003333
14	Aewol, Jeju, Jeju-do	KIOM201001003331
15	Aewol, Jeju, Jeju-do	KIOM201101003949
16	Aewol, Jeju, Jeju-do	KIOM201101003950
A. asiatica		
17	Icheon, Sangbuk, Ulju, Ulsan	KIOM201401009748
18	Sannae, Namwon, Jeollabuk-do	KIOM200801001156
19	Hyeol, Taebaek, Gangwon-do	KIOM201401009950
20	Danjibong, Gimcheon, Gyeongsangbuk-do	KIOM201501011463

Table S10: Accession numbers of cp genomes used for phylogenetic analysis.

Family	Taxon	GenBank accession number
Cimicifugeae	*Actaea heracleifolia*	NC_042253
	Actaea simplex	MN623225
	Actaea dahurica	MN623226
	Actaea biternata	MN623227
	Actaea asiatica	NC_041525
	Actaea vaginata	NC_041543
	Eranthis stellata	NC_041536
	Beesia calthifolia	NC_041531
	Anemonopsis macrophylla	NC_041527

Table S11: Selection of the best-fitting substitution model for 65 conserved cp genes using jModelTest.

	Model	f(a)	f(c)	f(g)	f(t)	kappa	titv	Ra	Rb	Rc	Rd	Re	Rf	pInv	Gamma
AIC	GTR+I+G	0.3	0.19	0.18	0.31	2.4	1.13	0.77	1.28	0.24	0.2	1.34	1	0.488	0.216

Model	-lnL*	K	AIC	delta	weight	cumWeight
GTR+I+G	110141.7	26	220335.4	0	0.545852	0.545852
TVM+I+G	110142.9	25	220335.7	0.37698	0.45208	0.997932
TIM1+I+G	110149.8	24	220347.6	12.21892	0.001213	0.999145
TPM1uf+I+G	110151.1	23	220348.3	12.9171	0.000855	1
GTR+I	110163.6	25	220377.2	41.85256	4.46E-10	1
TVM+I	110164.8	24	220377.6	42.25366	3.65E-10	1
GTR+G	110167.2	25	220384.3	48.94602	1.28E-11	1
TVM+G	110168.4	24	220384.8	49.4426	1.00E-11	1
TIM1+I	110171.7	23	220389.4	54.07156	9.90E-13	1
TPM1uf+I	110173.1	22	220390.1	54.77704	6.96E-13	1
TIM1+G	110175.3	23	220396.6	61.28992	2.68E-14	1
TPM1uf+G	110176.7	22	220397.4	62.01496	1.87E-14	1
TIM2+I+G	110344	24	220736	400.6717	5.40E-88	1
TPM2uf+I+G	110345.2	23	220736.4	401.0438	4.48E-88	1
TIM2+I	110365.9	23	220777.7	442.3458	4.82E-97	1
TPM2uf+I	110367.1	22	220778.1	442.7549	3.93E-97	1
TIM2+G	110368.2	23	220782.4	447.0647	4.55E-98	1
TPM2uf+G	110369.5	22	220782.9	447.5611	3.55E-98	1
TrN+I+G	110368.5	23	220783	447.6685	3.37E-98	1
HKY+I+G	110369.8	22	220783.7	448.3158	2.44E-98	1
TIM3+I+G	110368.4	24	220784.7	449.3737	1.43E-98	1
TPM3uf+I+G	110369.7	23	220785.4	450.0925	1.00E-98	1
TrN+I	110390.3	22	220824.5	489.1669	3.28E-107	1
HKY+I	110391.6	21	220825.2	489.8519	2.33E-107	1

*-lnL, negative log likelihood; K, number of estimated parameters; AIC, Akaike Information Criterion; delta, AIC difference; weight, AIC weight; cumWeight, cumulative AIC weight.

Table S12: Selection of the best-fitting substitution model for whole cp genomes using jModelTest.

	Model	f(a)	f(c)	f(g)	f(t)	kappa	titv	Ra	Rb	Rc	Rd	Re	Rf	pInv	Gamma
AIC	TVM+I+G	0.3	0.19	0.18	0.31	1.64	0.76	0.90	1.18	0.63	0.18	1.18	1	0.851	0.851

Model	-lnL*	K	AIC	delta	weight	cumWeight
TVM+I+G	289185.5398	25	578421.0796	0	0.684392	0.684392
GTR+I+G	289185.3138	26	578422.6277	1.54806	0.315608	1
TVM+I	289222.7514	24	578493.5027	72.42312	1.28E-16	1
GTR+I	289222.5696	25	578495.1392	74.05954	5.67E-17	1
TPM1uf+I+G	289347.6132	23	578741.2265	320.14686	2.07E-70	1
TIM1+I+G	289347.7098	24	578743.4196	322.33994	6.92E-71	1
TPM1uf+I	289387.4426	22	578818.8852	397.80558	2.84E-87	1
TIM1+I	289387.2983	23	578820.5966	399.51696	1.21E-87	1
TVM+G	289399.9797	24	578847.9595	426.87984	1.38E-93	1
GTR+G	289399.7773	25	578849.5546	428.47502	6.21E-94	1
TPM3uf+I+G	289552.8185	23	579151.6371	730.55744	1.57E-159	1
TIM3+I+G	289552.9781	24	579153.9561	732.87652	4.93E-160	1
TPM1uf+G	289555.0684	22	579154.1367	733.0571	4.51E-160	1
TIM1+G	289554.8922	23	579155.7845	734.70484	1.98E-160	1
TPM2uf+I+G	289571.4373	23	579188.8746	767.795	1.29E-167	1
HKY+I+G	289572.964	22	579189.9281	768.84844	7.62E-168	1
TIM2+I+G	289571.3345	24	579190.669	769.58934	5.26E-168	1
TrN+I+G	289575.0821	23	579196.1642	775.08454	3.37E-169	1
TPM3uf+I	289589.1743	22	579222.3486	801.26896	6.95E-175	1
TIM3+I	289588.9459	23	579223.8918	802.81222	3.21E-175	1
TPM2uf+I	289608.0302	22	579260.0603	838.98072	4.50E-183	1
TIM2+I	289607.8925	23	579261.785	840.70542	1.90E-183	1
HKY+I	289612.2148	21	579266.4296	845.35002	1.86E-184	1
TrN+I	289612.0516	22	579268.1031	847.0235	8.06E-185	1

*-lnL, negative log likelihood; K, number of estimated parameters; AIC, Akaike Information Criterion; delta, AIC difference; weight, AIC weight; cumWeight, cumulative AIC weight.
